# HIV-1 Drug Resistance Mutations: Potential Applications for Point-of-Care Genotypic Resistance Testing

**DOI:** 10.1371/journal.pone.0145772

**Published:** 2015-12-30

**Authors:** Soo-Yon Rhee, Michael R. Jordan, Elliot Raizes, Arlene Chua, Neil Parkin, Rami Kantor, Gert U. Van Zyl, Irene Mukui, Mina C. Hosseinipour, Lisa M. Frenkel, Nicaise Ndembi, Raph L. Hamers, Tobias F. Rinke de Wit, Carole L. Wallis, Ravindra K. Gupta, Joseph Fokam, Clement Zeh, Jonathan M. Schapiro, Sergio Carmona, David Katzenstein, Michele Tang, Avelin F. Aghokeng, Tulio De Oliveira, Annemarie M. J. Wensing, Joel E. Gallant, Mark A. Wainberg, Douglas D. Richman, Joseph E. Fitzgibbon, Marco Schito, Silvia Bertagnolio, Chunfu Yang, Robert W. Shafer

**Affiliations:** 1 Department of Medicine, Stanford University, Stanford, CA, United States of America; 2 Tufts University School of Medicine, Boston, MA, United States of America; 3 Division of Global HIV/AIDS, Centers for Disease Control and Prevention, Atlanta, GA, United States of America; 4 Medecins Sans Frontieres, Access Campaign, Geneva, Switzerland; 5 Institute of Infectious Diseases and Epidemiology, Tan Tock Seng Hospital, Singapore, Singapore; 6 Data First Consulting, Belmont, CA, United States of America; 7 Alpert Medical School, Brown University, Providence, RI, United States of America; 8 National Health Laboratory Service, Tygerberg, Coastal Branch, South Africa; 9 Division of Medical Virology, Stellenbosch University, Parow, South Africa; 10 National AIDS and Sexually Transmitted Infection (STI) Control Programme, Ministry of Health, Nairobi, Kenya; 11 UNC Project, Lilongwe, Malawi; 12 University of Washington and Seattle Children’s Research Institute, Seattle, WA, United States of America; 13 Institute of Human Virology, Abuja, Nigeria; 14 Amsterdam Institute for Global Health and Development (AIGHD), Department of Global Health, Academic Medical Center of the University of Amsterdam, Amsterdam, Netherlands; 15 Lancet Laboratories and BARC-SA, Johannesburg, South Africa; 16 Department of Infection, University College London, London, United Kingdom; 17 Chantal BIYA International Reference Centre for Research on HIV/AIDS Prevention and Management, Yaoundé, Cameroon; 18 Faculty of Medicine and Biomedical Sciences (FMBS) of the University of Yaounde 1, Yaounde, Cameroon; 19 Division of HIV/AIDS Prevention, Centers for Disease Control and Prevention, Atlanta, Georgia, United States of America; 20 National Hemophilia Center, Tel Hashomer, Israel; 21 Department of Haematology and Molecular Medicine, University of Witwatersrand, Johannesburg, South Africa; 22 National Health Laboratory Services, Johannesburg, South Africa; 23 Virology Laboratory CREMER-IMPM, Yaoundé, Cameroon; 24 Africa Centre for Health and Population Studies, School of Laboratory Medicine and Medical Sciences, University of KwaZulu-Natal, Durban, South Africa; 25 Virology, Department of Medical Microbiology, University Medical Center Utrecht, Utrecht, the Netherlands; 26 Southwest CARE Center, Santa Fe, NM, United States of America; 27 McGill University AIDS Centre, Jewish General Hospital, Montreal, Quebec, Canada; 28 Department of Pathology, University of California San Diego, La Jolla, CA, United States of America; 29 Veterans Affairs San Diego Healthcare System, San Diego, CA, United States of America; 30 Drug Development and Clinical Sciences Branch, Division of AIDS, National Institute of Allergy and Infectious Diseases, National Institutes of Health, Bethesda, MD, United States of America; 31 HJF-DAIDS, A Division of The Henry M. Jackson Foundation for the Advancement of Military Medicine, Inc., Bethesda, MD, United States of America; 32 HIV Department WHO, Geneva, Switzerland; University of British Columbia, CANADA

## Abstract

The increasing prevalence of acquired and transmitted HIV-1 drug resistance is an obstacle to successful antiretroviral therapy (ART) in the low- and middle-income countries (LMICs) hardest hit by the HIV-1 pandemic. Genotypic drug resistance testing could facilitate the choice of initial ART in areas with rising transmitted drug resistance (TDR) and enable care-providers to determine which individuals with virological failure (VF) on a first- or second-line ART regimen require a change in treatment. An inexpensive near point-of-care (POC) genotypic resistance test would be useful in settings where the resources, capacity, and infrastructure to perform standard genotypic drug resistance testing are limited. Such a test would be particularly useful in conjunction with the POC HIV-1 viral load tests that are currently being introduced in LMICs. A POC genotypic resistance test is likely to involve the use of allele-specific point mutation assays for detecting drug-resistance mutations (DRMs). This study proposes that two major nucleoside reverse transcriptase inhibitor (NRTI)-associated DRMs (M184V and K65R) and four major NNRTI-associated DRMs (K103N, Y181C, G190A, and V106M) would be the most useful for POC genotypic resistance testing in LMIC settings. One or more of these six DRMs was present in 61.2% of analyzed virus sequences from ART-naïve individuals with intermediate or high-level TDR and 98.8% of analyzed virus sequences from individuals on a first-line NRTI/NNRTI-containing regimen with intermediate or high-level acquired drug resistance. The detection of one or more of these DRMs in an ART-naïve individual or in a individual with VF on a first-line NRTI/NNRTI-containing regimen may be considered an indication for a protease inhibitor (PI)-containing regimen or closer virological monitoring based on cost-effectiveness or country policy.

## Introduction

The global scale-up of antiretroviral therapy (ART) has dramatically reduced HIV-1-associated mortality, mother-to-child HIV-1 transmission, and adult HIV-1 incidence [1–4]. These public health accomplishments are the result of the widespread administration of standardized first-line regimens containing two nucleoside reverse transcriptase inhibitors (NRTIs) plus a non-nucleoside RT inhibitor (NNRTI), followed by a ritonavir-boosted lopinavir (LPV/r)-containing regimen in those individuals who subsequently develop virological failure (VF) [5, 6]. However, the margin of long-term ART success is compromised by the development of acquired drug resistance (ADR) and transmitted drug resistance (TDR) [7, 8].

Between 10% and 30% of individuals receiving a first-line NRTI/NNRTI-containing treatment regimen will develop VF at some point during their treatment [9–11]; the majority of these individuals are expected to acquire NRTI- and/or NNRTI-resistant viruses [7, 11–13]. As the number of individuals with ADR has increased so has the proportion of newly infected individuals with TDR [7, 14–16]. In many regions, the proportion of individuals with transmitted NNRTI resistance has been increasing since ART scale-up [7, 14, 15]. In recent studies, TDR levels above five percent were reported in about one-fourth of the surveys conducted in Sub-Saharan Africa and South/Southeast Asia and more than one-half of the surveys conducted in the Latin America/Caribbean region [7, 14, 15, 17, 18].

In upper-income countries, HIV-1 genotypic resistance testing is used to guide the selection of initial ART and subsequent treatments in individuals with VF. However, in the low- and middle-income countries (LMICs), the resources and capacity to perform standard genotypic resistance testing for individual management are limited or concentrated in a few central laboratories. A point-of-care (POC) genotypic resistance test would avoid the logistical challenges and delays associated with centralized genotypic resistance testing. Even in the context of a public health approach to ART, where few standardized regimens are available, a reliable and inexpensive POC genotypic resistance test would enable HIV-1 care providers to optimize HIV treatment management and make informed treatment decisions for three categories of individuals: (1) ART-naïve individuals starting therapy; (2) individuals with VF on an initial NRTI/NNRTI-containing regimen; and (3) individuals with persistently detectable viremia on a first- or second-line protease inhibitor (PI)-containing regimen. The objective of this study was to develop recommendations on the most useful drug-resistance mutations (DRMs) for genotypic drug resistance testing to encourage manufacturers developing POC diagnostic assays.

## Materials and Methods

### Conceptual design

As the choice of DRMs for a POC genotypic resistance test depends on the proportions of DRMs in different populations and on expert opinion of the clinical significance of DRMs in LMIC settings, the senior author and the main funder agreed that this paper should be written by a group of experts in the areas of drug resistance, assay development, and public health. The senior author joined with HIV drug resistance experts at the World Health Organization (WHO), U.S. National Institutes of Health (NIH), and U.S. Centers for Disease Control and Prevention (CDC) to assemble a group of experts who reviewed successive drafts of this manuscript.

Because NRTIs, NNRTIs, and PIs are the ARV classes used in most LMICs, we sought to identify the NRTI, NNRTI, and PI-associated DRMs with the greatest sensitivity and specificity for ARV selection pressure and the greatest effect on *in vitro* and *in vivo* ARV susceptibility. The frequency of mutations was analyzed in publicly available datasets comprising individuals with TDR, VF on a first-line NRTI/NNRTI-containing regimen, and VF on an initial boosted PI-containing regimen.

### Mutation classification

The Stanford HIV Drug Resistance Database (HIVDB) mutation penalty scoring system was used to identify major NRTI, NNRTI, and PI-resistance mutations and to characterize the level of resistance in clinical isolates. HIVDB has an online genotypic resistance interpretation program to help clinicians and laboratories interpret HIV-1 genotypic resistance tests. The program accepts submitted RT, protease and/or integrase sequences and returns a list of penalty scores for each DRM in the sequence and an estimate of reduced susceptibility for each ARV obtained by adding the penalty scores for each DRM. The DRM penalty scores (http://hivdb.stanford.edu/DR/) are based upon the sensitivity and specificity of a mutation for selective ARV drug pressure and the effect of a mutation on *in vitro* susceptibility and virological response to therapy. The DRM penalty scores also reflect consensus about the clinical significance of a DRM as reflected by experts such as the IAS-USA Drug Resistance Mutations Group [19].

An HIVDB penalty score of 15 to 29 predicts low-level resistance; a score of 30 to 59 predicts intermediate resistance; and a score of 60 or above predicts high-level resistance. In this analysis, NRTI and PI DRMs with a score of 30 or more and NNRTI DRMs with a score of 60 or more are referred to as major DRMs. A lower score cut-off is used for the NRTIs and PIs because high-level NRTI and PI resistance usually results from the accumulation of multiple DRMs associated with low-level and intermediate resistance rather than from a single DRM associated with high-level resistance. S1, S2 and S3 Tables contain the HIVDB DRM penalty scores and summarize information that were considered in developing the HIVDB scoring system.

### Analyses

To identify the most common major NRTI and NNRTI DRMs associated with TDR, we used a dataset of sequences from more than 50,000 individuals included in a recently published meta-analysis of 287 studies of which 151 studies were conducted in Sub-Saharan Africa and the LMICs of South/Southeast Asia [16].

To identify the most common major NRTI and NNRTI DRMs associated with VF on a first-line NRTI/NNRTI-containing regimen, we used a dataset of sequences from 4,926 individuals from 68 published studies available in HIVDB through June 2014 for which RT sequences were available. Each study contained sequences from five or more individuals receiving a first-line regimen comprising emtricitabine (FTC) or lamivudine (3TC) plus abacavir (ABC) stavudine (d4T), tenofovir (TDF), or zidovudine (AZT) plus efavirenz (EFV) or nevirapine (NVP).

To identify the most common major PI-associated DRMs, we used a dataset of sequences from 1,214 previously PI-naïve individuals with VF on an LPV/r-containing regimen. An insufficient number of published sequences were available from previously PI-naïve individuals receiving ritonavir-boosted atazanavir (ATV/r) or darunavir (DRV/r) to perform a similar analysis. Therefore, for these PIs, we used data from published studies whether or not the underlying sequences were publicly available.

For the analysis of individuals with TDR or VF on a first-line NRTI/NNRTI-containing regimen we examined the sensitivity of sets of mutations for identifying individuals having viruses with intermediate or high-level resistance to the NRTIs 3TC, ABC, AZT, FTC, or TDF; intermediate or high-level resistance to the NNRTIs NVP or EFV; and intermediate or high-level resistance to one of the preceding NRTIs or NNRTIs. For the RT and protease, different mutations at the same position (e.g., M184V and M184I) were treated as separate DRMs despite the fact that some POC assays may be designed to detect multiple amino acids at the same position. Tier 1 POC DRMs were defined as DRMs that the authors believed should ideally be included in an initial version of a POC genotypic resistance test. Non-tier 1 POC DRMs were defined as DRMs that might be useful in subsequent versions of a POC test contingent upon changes in ARV treatment strategies and the molecular epidemiology of HIV-1 drug resistance.

## Results

### Most common major NRTI- and NNRTI-resistance mutations in individuals with TDR

**Fig 1:**
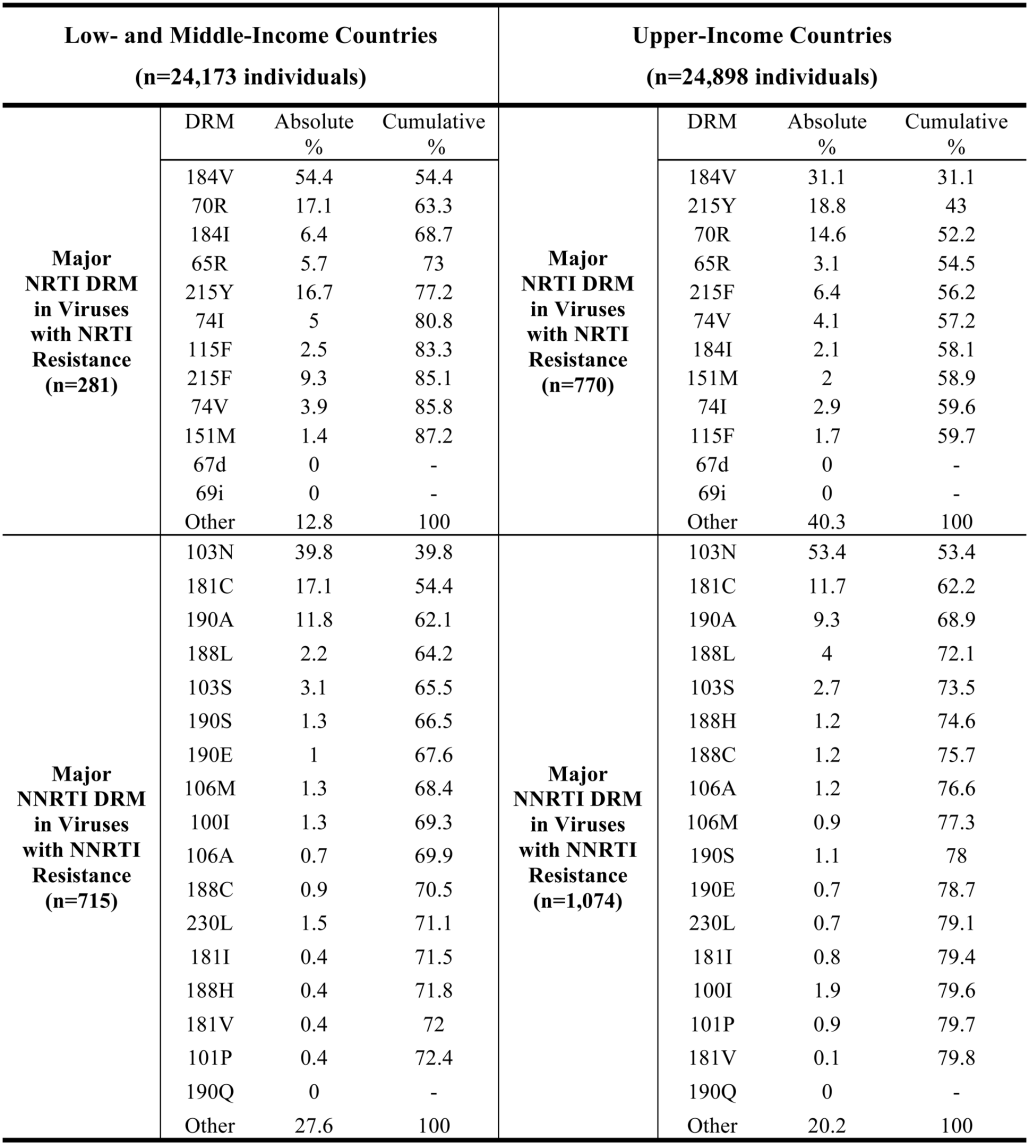
Prevalence of major NRTI and NNRTI resistance mutations in individuals with transmitted drug resistance. Absolute and cumulative prevalence of each major nucleoside (NRTI) and nonnucleoside RT Inhibitor (NNRTI) drug-resistance mutation (DRM) in individuals with intermediate or high-level transmitted NRTI or NNRTI resistance from a meta-analysis of 287 studies published between 2000 and 2013 are shown. Low- and middle- income countries include Countries of Sub-Saharan Africa, South / Southeast Asia, and Latin America and Caribbean. Upper-Income Countries: Countries of North America and Europe, and upper-income countries in Southeast Asia. Major NRTI DRMs include those with an HIVDB score ≥30. There were no insertions or deletions between codons 67 and 70. Major NNRTI DRMs include those with an HIVDB score ≥60. Absolute %: number of individuals with DRM / number of individuals with intermediate or high-level transmitted NRTI or NNRTI resistance. Cumulative %: number of individuals with one or more of the preceding major DRMs in the list / number of individuals with intermediate or high-level transmitted NRTI or NNRTI resistance.

Fig 1 shows the absolute and cumulative prevalence of the major NRTI and NNRTI DRMs in RT sequences from a recently published individual-level meta-analysis of more than 50,000 ARV-naïve individuals in 287 published studies [16]. M184V was the most common transmitted major NRTI-associated DRM, occurring in 54% of viruses from individuals with intermediate or high-level NRTI TDR in LMICs and 31% of viruses from individuals with intermediate or high-level NRTI TDR in upper-income countries. M184I, K65R, L74V/I, Y115F and the TAMs K70R and T215Y/F were the next most common transmitted major NRTI DRMs. The TAMs M41L, D67N/E/G and K219Q/E/N/R and the T215 revertant mutations (T215C/D/E/S/I/V) were the most common non-major transmitted NRTI DRMs. Subtype was not a major determinant of which NRTI DRMs occurred in TDR isolates in LMICs.

K103N, Y181C and G190A were the three most common NNRTI DRMs in all regions and subtypes, occurring in more than 60% of viruses with intermediate or high-level NNRTI TDR. K103S, V106M, Y188L and G190S/E accounted for most of the remaining transmitted major NNRTI DRMs in LMICs. A98G and K101E were the most common non-major transmitted NNRTI DRMs. V106M was significantly more common in subtype C viruses and was the fourth most common NNRTI DRM in this subtype.

### Most common major NRTI- and NNRTI-resistance mutations in individuals with first-line VF

S4 Table summarizes the number of sequences and corresponding individuals receiving NRT/NNRTI first-line ART according to regimen and HIV-1 subtype. Fifty-five percent, 27%, 16% and 2% of individuals received a d4T-, AZT-, TDF- or ABC-containing regimen, respectively. Fifty-four percent received EFV and 46% received NVP. The most common subtypes were C (46%), circulating recombinant form (CRF) 01_AE (15%), B (11%), A (8%), G (8%) and CRF02_AG (7%). Seventy-three percent of individuals had one or more major NRTI DRM and one or more major NNRTI DRM. Nine percent had a major NNRTI DRM but no major NRTI DRM; 2% percent had a major NRTI DRM but no major NNRTI DRM; and 16% had no major NRTI or NNRTI DRM.

**Fig 2:**
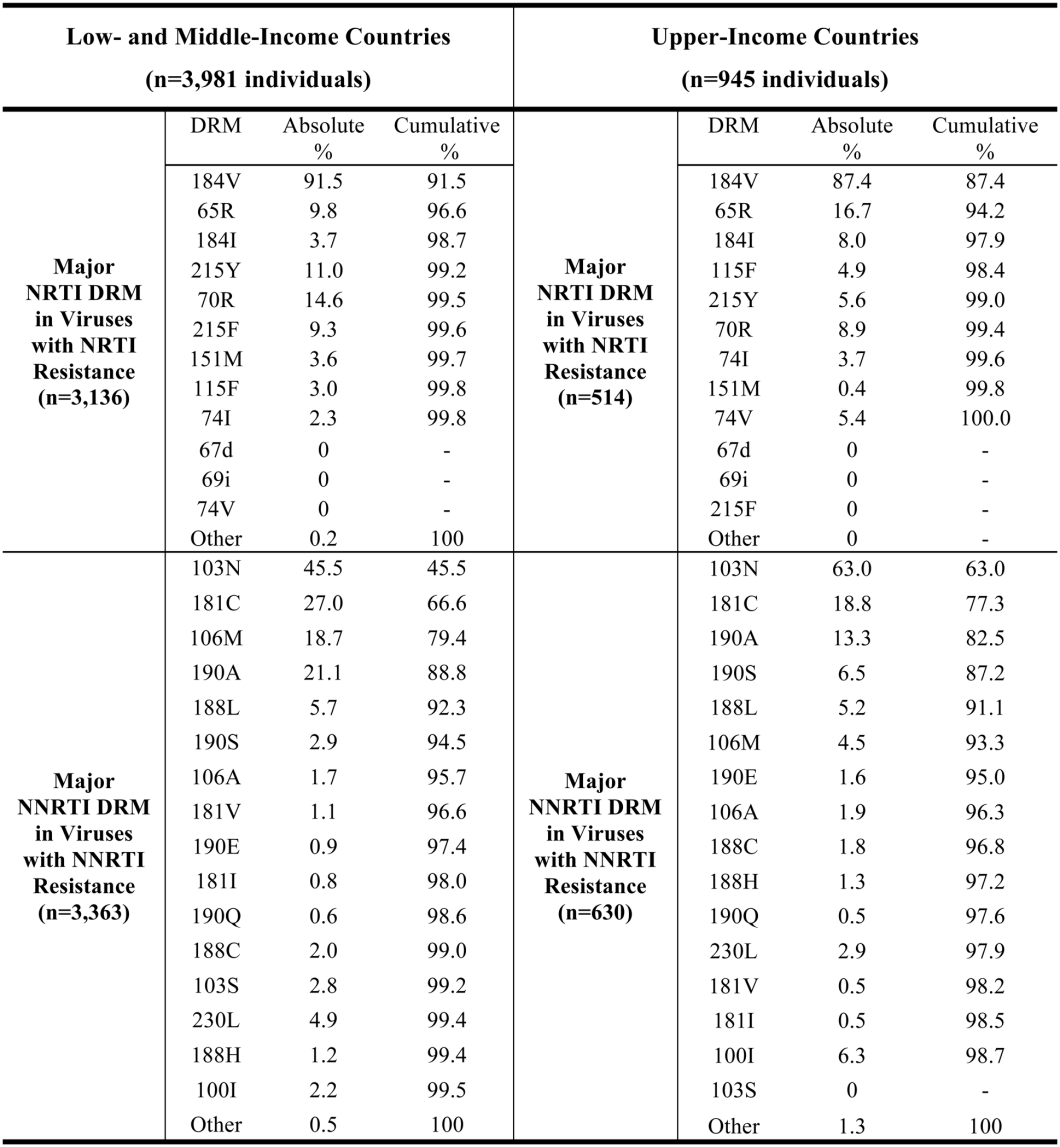
Prevalence of major NRTI and NNRTI resistance mutations in individuals with acquired drug resistance. Absolute and cumulative prevalence of each major nucleoside (NRTI) and nonnucleoside RT inhibitor (NNRTI) drug-resistance mutation (DRM) in 4,926 individuals with virological failure and intermediate or high-level acquired NRTI or NNRTI resistance while receiving a first-line NRTI/NNRTI-containing regimen are shown. Regimens include four AZT/d4T-containing regimens—AZT/d4T+3TC+EFV/NVP (n = 4,020), four TDF-containing regimens—TDF+3TC/FTC+EFV/NVP (n = 772), and two ABC-containing regimens—ABC+3TC+NVP/EFV (n = 134). Low- and Middle-Income Countries: Countries of Sub-Saharan Africa, South / Southeast Asia, and Latin America and Caribbean; Upper-Income Countries: Countries of North America and Europe, and upper-income countries in Southeast Asia. NRTI DRM with an HIVDB score ≥30. There were no insertions or deletions between codons 67 and 70. NNRTI DRMs with an HIVDB score ≥60. Absolute %: number of individuals with DRM / number of individuals with intermediate or high-level NRTI or NNRTI resistance. Cumulative %: number of individuals with one or more of the preceding DRMs in the list / number of individuals with intermediate or high-level NRTI or NNRTI resistance.

Fig 2 shows that in viruses from individuals in LMICs with intermediate or high-level NRTI resistance following VF on a first-line NRTI/NNRTI-containing regimen, the most common major DRMs were M184V (91.5%) and M184I (3.7%), K65R (9.8%), and the TAMs K70R (14.6%), T215Y (11.0%) and T215F (9.3%). About one-half of the viruses with K65R did not have M184V, making K65R the second largest contributor to the cumulative proportion of viruses with a major NRTI DRM. K65R also occurred in 48% of 467 individuals with VF on a first-line TDF-containing regimen (S5 Table). The TAMs nearly always occurred in combination with M184V and contributed less to the cumulative proportion of viruses with a major NRTI DRM than did K65R.

The spectrum of DRMs in 712 children was similar to adults with the exception that L74V/I occurred more often in children because a higher proportion of children received an ABC-containing regimen (S6 and S7 Tables). Indeed, among both adults and children receiving ABC, L74V/I were the second most common major NRTI DRMs associated with acquired NRTI resistance after M184V [20, 21]. L74V/I rarely occurred in the absence of M184V.

Fig 2 shows that the most common NNRTI DRMs in viruses from individuals in LMICs with intermediate or high-level resistance on a first-line NRTI/NNRTI-containing regimen were K103N (45.5%), Y181C (27.0%), G190A (21.0%), and V106M (18.7%). One or more of these four DRMs occurred in 88.8% of viruses with intermediate or high-level acquired NNRTI resistance. V106M was the second-most common NNRTI DRM in subtype C viruses, occurring in 33% of individuals with a major NNRTI DRM. The two next most common NNRTI DRMs—Y188L and G190S –accounted for an additional 5.7% of viruses with acquired intermediate or high-level NNRTI resistance.

### The sensitivity of combined NRTI and NNRTI-resistance mutations for detecting TDR and ADR

**Fig 3:**
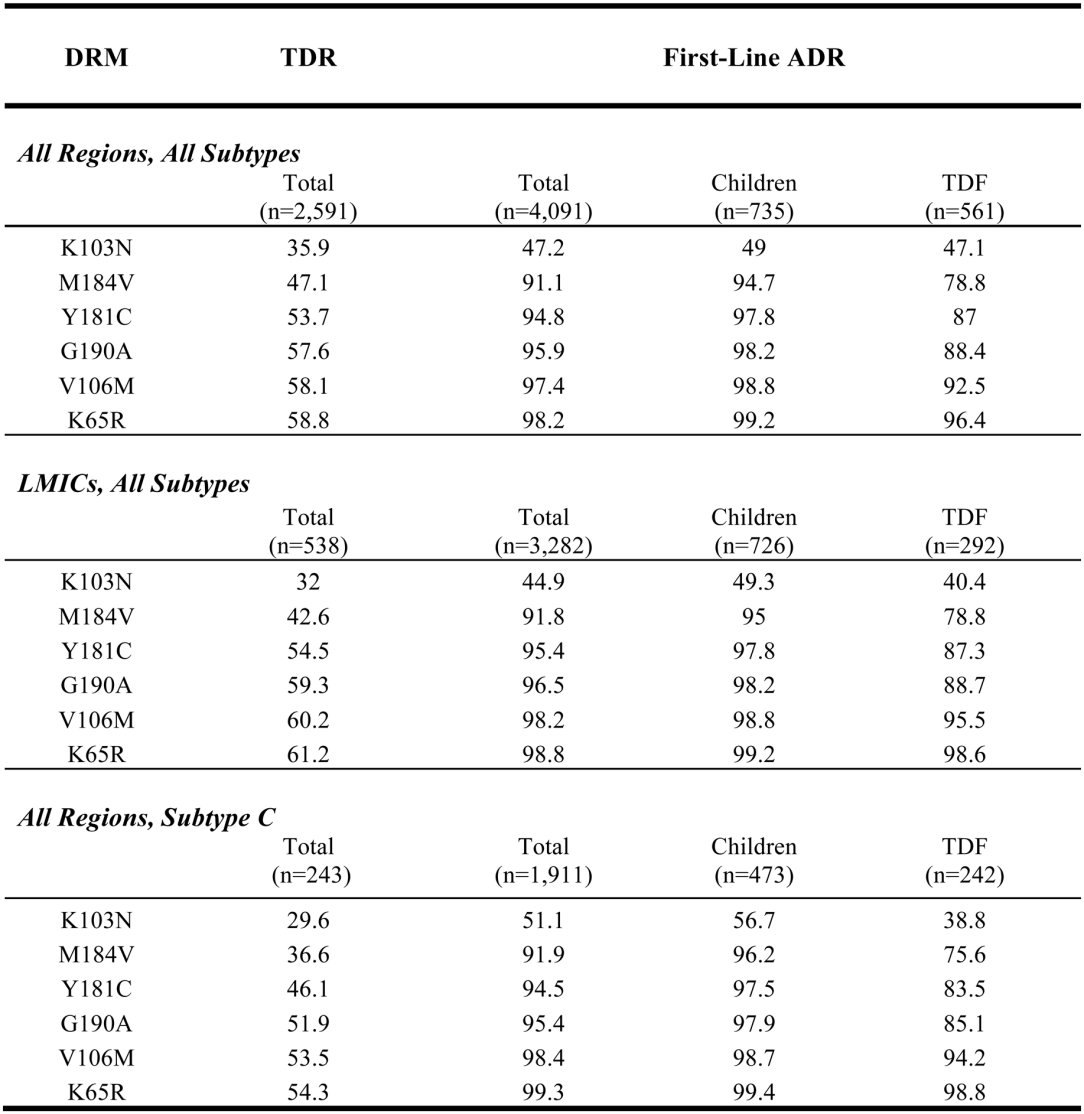
Sensitivity of Six Tier 1 RTI resistance mutations for detecting transmitted or acquired drug resistance. Cumulative prevalence or sensitivity of the Six Tier 1 RT inhibitor (RTI) drug-resistance mutations (DRMs) for detecting transmitted or acquired drug resistance in viruses from individuals with intermediate or high-level NRTI or NNRTI resistance is shown. Transmitted drug resistance (TDR) and acquired drug resistance (ADR) were defined as being associated with ≥ intermediate NRTI or NNRTI resistance. Major NRTI-associated DRMs (HIVDB score ≥30) included K65R, D67 deletion, T69 insertion, K70R, L74V/I, Y115F, Q151M, M184I/V, and T215F/Y. Major NNRTI-associated DRMs (HIVDB score ≥60) included: L100I, K101P, K103N/S, V106A/M, Y181C/I/V, Y188L/H/C, G190A/S/E/Q, and M230L. Abbreviations: LMICs (Low- and Middle-Income Countries), TDF (tenofovir).

Of the six DRMs with the highest cumulative sensitivity for detecting intermediate or high-level resistance to an NRTI or NNRTI on a first-line NRTI/NNRTI-containing regimen (K65R, M184V, K103N, V106M, Y181C, G190A), all but K65R and V106M were also the most common DRMs occurring in individuals with TDR. In LMICs, this set of six DRMs was 98.8% sensitive for detecting ADR on a first-line NRTI/NNRTI regimen and 61.2% sensitive for detecting TDR in ART-naïve individuals (Fig 3). No significant differences in sensitivity were observed for the subset of children with ADR on a first-line NRTI/NNRTI-containing regimen or the subset of adult individuals with ADR on a first-line TDF-containing regimen (Fig 3). Based on the clinical significance of these DRMs and their sensitivity and specificity for ADR and, to a lesser extent TDR, these DRMs were classified as tier 1 POC DRMs.

Y188L and G190S were the most common major non-tier 1 NNRTI DRMs associated with ADR on a first-line NRTI/NNRTI-containing regimen and among the most common non-tier 1 NNRTI DRMs associated with TDR. K70R, L74V/I, Y115F, M184I, and T215Y/F were the most common major non-tier 1 DRMs associated with ADR on a first-line NRTI/NNRTI-containing regimen and with TDR. Compared to an assay that detected just tier 1 RTI DRMs, an assay that detected tier 1 and the aforementioned nine additional non-tier 1 DRMs would have an increased sensitivity for detecting intermediate or high-level TDR of 67.9% (compared with 61.2%) and for detecting dual NRTI/NNRTI resistance in individuals with ADR of 94.2% (compared with 84.9%). However, an assay with tier 1 and the nine non-tier 1 RTI DRMs would not have a meaningful effect on the sensitivity for detecting intermediate or high-level ADR resistance to either NRTIs or NNRTIs (99.5% compared with 98.8%).

### DRMs emerging in previously PI-naïve individuals receiving LPV/r, ATV/r, and DRV/r

Table 1 shows the most common major LPV-associated DRMs in published protease sequences from 1,214 previously PI-naïve individuals with VF on an LPV/r-containing regimen. Of these 1,214 individuals, 203 (17%) had viruses with predicted intermediate or high-level LPV resistance. The most common major PI DRMs were V82A, I76V, I84V and L47A. One or more of these four DRMs occurred in 88% of viruses with intermediate or high-level LPV/r resistance. The next two most common major LPV DRMs—I50V and V82F –accounted for an additional 4% of viruses with predicted intermediate or high-level LPV resistance. The remaining 8% of viruses with predicted intermediate or high-level LPV resistance had a combination of two or more PI DRMs with lower mutation scores, including V32I, M46I, I54M/L/V, I47V, V82S/T/M and L90M. The most common subtypes of these 203 viruses were C (49%), CRF01_AE (14%), CRF01_AG (12%), B (8%), G (7%) and A (5%). Overall 170 (84%) of the 203 LPV-resistant viruses had predicted intermediate or high-level cross-resistance to ATV/r; 36 (18%) had predicted intermediate or high-level cross-resistance to DRV/r.

**Table 1:** Absolute and Cumulative Prevalence of Major Lopinavir-Associated Mutations in 203 Viruses with Intermediate or High-Level Lopinavir (LPV) Resistance from 1,214 Previously PI-Naïve Individuals with Virological Failure on a Ritonavir-Boosted LPV (LPV/r)-Containing Regimen.

DRM	Prevalence of Major LPV/r DRMs (n = 203 Viruses with Intermediate or High-Level LPV Resistance)	
	Absolute %^a^	Cumulative %^b^
V82A	59.6	59.6
L76V	32.5	74.9
I84V	15.3	82.8
I47A	8.4	88.2
V82F	2.5	90.1
I50V	4.9	91.6
Other ^c^	8.4	100

^a^Absolute %: number of individuals with major DRM / number of individuals with intermediate or high-level LPV resistance.

^b^Cumulative %: number of individuals with one or more of the preceding major LPV/r DRMs in the list / number of individuals with intermediate or high-level LPV resistance.

^c^Other includes viruses with intermediate or high-level resistance arising from an accumulation of mutations with an HIVDB penalty score <30 including: M46I/I54V/V82S (n = 4), I54V/V82M (n = 3), I54V/L90M (n = 1), V32I/M46I/I47V/I54M/L90M (n = 1), I54V/V82T/L90M (n = 1), M46I/I54V/V82T (n = 1), I54V/V82T (n = 1), I54V/V82S/V82T (n = 1), L90M (n = 1), M46I/L90M (n = 1), M46I/I47V/I54V/V82S (n = 1).

Few protease sequences are available from PI-naïve individuals with VF on ATV/r- or DRV/r-containing regimens. Published reports of aggregated data indicate that I50L and N88S are the main DRMs developing in PI-naïve individuals with VF on an ATV- or ATV/r-containing regimen [22–24]. These DRMs do not confer cross-resistance to LPV or DRV [25]. In fact, I50L is associated with increased susceptibility to LPV, DRV and other PIs [26].

## Discussion

### Clinical scenarios in which POC genotypic resistance testing would be most useful

In regions where surveillance indicates elevated levels of drug resistance in individuals beginning ART, pre-therapy POC genotypic resistance testing would identify those individuals who should receive standard first-line therapy and those who should instead receive a boosted PI-containing regimen. Genotypic resistance testing would also be particularly useful in the management of the increasing proportion of individuals presenting for care with reported or unreported prior exposure to ARVs and to ensure that HIV-1-infected pregnant women with drug-resistant viruses receive the optimal regimen for themselves and to prevent mother-to-child transmission.

POC genotypic resistance testing would also be useful in the management of patients receiving an initial NRTI/NNRTI containing regimens. As there is an expanding pipeline of POC and near-POC assays for measuring HIV-1 virus load [27–30], coupling a POC genotypic resistance test with a POC viral load test will help HIV care providers determine in a single visit which individuals require further adherence support and which individuals should switch regimens [15, 31, 32].

The current WHO algorithm for the management of VF includes an adherence intervention after the first detected VF and a repeated virus load test three months thereafter. If the second virus load test confirms VF, a switch to second-line ART is recommended. Despite this recommendation, HIV care providers are often uncertain as to when to switch individuals to the more costly second-line ARVs out of concerns that VF resulted from nonadherence. A POC genotypic resistance test would provide confidence and empower care providers to make timely treatment decisions. Same day treatment decisions would eliminate the risk of loss to follow-up between the first and second virus load visits, minimize the risk of specimen mishandling, and allow adherence support strategies to be informed by the results of genotypic resistance testing.

### Selection of DRMs for POC genotypic resistance testing

One or more of the six tier 1 RT inhibitor DRMs was present in 61.2% of ARV-naïve individuals with intermediate or high-level resistance and in 98.8% of ARV-experienced individuals with intermediate or high-level resistance following VF on a first-line NRTI/NNRTI-containing regimen. These six DRMs include two NRTI-associated DRMs (K65R and M184V) and four NNRTI-associated DRMs (K103N, V106M, Y181C, and G190A). These DRMs are specific indicators of drug resistance in that they each cause clinically significant reduced susceptibility to one or more of the ARVs used in LMICs and rarely occur in the absence of selective ARV pressure. A POC genotypic resistance assay that reliably detected these six DRMs would therefore be moderately sensitive and highly specific for detecting TDR and highly sensitive and specific for detecting NRTI or NNRTI resistance in individuals with VF on a first-line NRTI/NNRTI-containing regimen.

In an ART-naïve individual, the presence of each of the tier 1 DRMs except K65R may be considered an indication for starting an initial PI-containing regimen or closer virological monitoring based on cost-effectiveness or country policy. The presence of K65R would be an indication for using an AZT/3TC nucleoside backbone. In individuals with VF on a 1^st^-line NRTI/NNRTI-containing regimen, the presence of a tier 1 DRM indicates that the regimen has reduced antiviral activity. Although the presence of a tier 1 DRM in individuals with VF on a first-line NRTI/NNRTI regimen would not preclude a virological response to continued therapy with improved adherence [33–35], continued therapy is expected to result in a higher rate of immunological and clinical deterioration than would occur if the individual is switched to a second-line PI-based therapy.

The most common additional major NNRTI-resistance mutations included Y188L and G190S, both of which are usually two-base pair mutations that cause very high levels of resistance to NVP and EFV. The next most common additional major NRTI-resistance mutations include M184I, which often precedes M184V in individuals receiving 3TC and FTC, L74V/I, which occurs most commonly in individuals receiving ABC, and the TAMs K70R and T215Y/F. A POC genotypic resistance assay able to detect additional RTI-associated DRMs would have increased sensitivity for TDR and for detecting dual NRTI/NNRTI resistance in individuals with VF on a first-line NRTI/NNRTI-containing regimen. However, considering the technical challenges associated with the inclusion of each additional DRM in a point mutation assay, the inclusion of non-tier 1 RTI-associated mutations in a POC assay was considered a lower priority.

PI DRMs develop much less often in individuals receiving a potent ritonavir-boosted PI-containing regimen such than do NRTI and NNRTI DRMs in individuals receiving NRTI/NNRTI-containing regimens [20, 24, 36–39]. The reduced risk of resistance associated with boosted PIs is likely due to the narrow drug concentration range in which PI levels are both low enough to allow virus replication and high enough to exert selective drug pressure [40]. Indeed, most individuals without PI DRMs who experience VF while on an initial PI-containing regimen achieve virologic suppression with improved adherence [41]. The possibility that mutations outside of protease may also be primary causes of VF is an area of active investigation [42, 43].

Our analysis suggests that the four mutations V82A, L76V, I84V, and I47A would have a sensitivity approaching 90% for detecting intermediate or high-level LPV resistance and that I50L and N88S are the most common major PI-associated DRMs to develop in individuals with VF and intermediate or high-level ATV resistance on an initial ATV/r-associated regimen. However, considering the recent addition of ATV/r to the WHO treatment guidelines and the absence of recommended third-line treatment options [44], it would be useful to have additional data on the genetic mechanisms of PI resistance in LMICs before making firm recommendations on an optimal set of POC DRMs for use in individuals receiving an initial boosted PI-containing regimen.

### Limitations of POC genotypic resistance testing

Although the analyses in this manuscript show that a carefully chosen set of DRMs provides useful information for the most common treatment scenarios in LMICs, a POC genotypic resistance test would not detect many DRMs that would be detected by standard sequencing. The added information provided by sequencing would be most useful in individuals with VF who have received multiple ARV regimens and in situations in which the extent of drug resistance was important. The usefulness of a POC assay that detected a limited set of mutations would also depend on active surveillance systems to identify emerging trends in the prevalence of transmitted and acquired clinically significant DRMs.

Standard genotypic resistance testing using dideoxyterminator Sanger sequencing is becoming less expensive and can be performed for under $100 if the sequence is limited to the most relevant part of the RT gene [45, 46]. Next-generation sequencing using the Illumina MiSeq platform can be performed for an even lower cost if large numbers of samples are multiplexed in a single sequencing reaction [47]. The extensive batching of samples required to obtain this cost reduction, however, is a disadvantage in settings where timeliness is important.

Although many allele-specific point mutation assays for HIV-1 drug resistance have been developed for research purposes, only a few have been developed and studied for their reliability and applicability in routine individual management [48–50]. The main challenge in developing such assays is that HIV-1 sequence variability at and surrounding each DRM complicates the hybridization steps required for mutation detection. Overall 42 codons at positions 65, 103, 106, 181, 184, and 190 occur in 1% or more sequences of the seven most common subtypes (A, B, C, D, G, CRF01_AE, and CRF02_AG). Thirteen of these 42 codons encode the six major DRMs proposed in this manuscript [51].

The sequence variability surrounding each drug-resistance position may present a more formidable challenge than the variability at the codons of interest. Flanking sequence variability interfered with the clinical uptake of two previously developed hybridization-based assays, the Affymetrix GeneChip HIV PRT 440 and the Innogenetics INNO-LiPA HIV-1 RT assays [52, 53]. It has also influenced the design of newer point mutation assays that are currently being developed for clinical use in LMICs [48–50]. S8 and S9 Tables indicate the extent of this variability at and surrounding codon 103 for sequences from a combination of 26,358 ARV-naïve and ARV-experienced individuals in six LMIC regions obtained from HIVDB [51]. It is beyond the scope of this manuscript, however, to describe the various strategies being used to maximize the stringency for DRM discrimination while accommodating for one or more flanking sequence mismatches.

### Evolution of ARV treatment strategies

The extent to which ATV/r will be used for second-line therapy and the potential availability of DRV/r and the integrase strand transfer inhibitors (INSTIs) are key areas of uncertainty. Although LPV/r and ATV/r-containing regimens are equally effective for initial ART [54, 55], ATV/r-containing regimens may be less efficacious for second-line therapy. ATV/r has a lower genetic barrier to resistance than LPV/r and ATV/r monotherapy has been less effective than LPV/r for regimen simplification [56–58] suggesting that ATV/r may be less effective than LPV/r in treating individuals with extensive NRTI resistance. Therefore, the extent of NRTI resistance following initial therapy may have greater implications for the use of ATV/r- than for LPV/r-containing second-line regimens.

However, if ATV/r-containing second-line regimens prove effective, their use would have favorable implications for both POC testing and third-line treatment. I50L and N88S are the most commonly occurring major DRMs in PI-naïve individuals receiving ATV/r. Identifying clinically relevant ATV resistance would therefore be simpler than identifying the more complex patterns of DRMs associated with LPV resistance. In addition, most individuals with VF on a second-line ATV/r-containing regimen are expected to have viruses that are fully susceptible to LPV and DRV making it possible to create a highly effective third-line regimen using these PIs.

Although the NNRTI rilpivirine (RPV) has recently been approved in upper-income countries for use in a fixed-dose combination with TDF and FTC, further studies would be necessary before it could be considered a standard first-line treatment option in LMICs. In particular, RPV is approved only for individuals with plasma HIV-1 RNA levels below 100,000 copies//ml. A POC test for RPV resistance would also require a different set of NNRTI-associated DRMs than those described here because K101E, E138K, and Y181C appear to be the DRMs occurring most commonly in individuals receiving first-line RPV-containing regimens [19, 59].

It is difficult to predict how the introduction of INSTIs will influence the development of POC genotypic resistance testing strategies because such strategies depend on which INSTIs will be introduced and on whether they will be used for first-, second- or third-line therapy [60]. However, if INSTIs will be used beyond the first line of therapy and in combination with NRTIs, it may become important to identify the NRTI DRMs most likely to increase the risk of VF on an NRTI/INSTI-containing regimen.

Ongoing population-level surveillance of both ARV-naïve and experienced individuals using standard genotypic resistance testing will be important to detect changing patterns in the molecular epidemiology of HIV-1 drug resistance resulting either from changing ARV selection pressures and/or transmission dynamics. The cost-effectiveness of POC genotypic resistance testing will depend on the evolving molecular epidemiology of ADR and TDR, the clinical uptake of POC virus load testing, and the number of available regional treatment options. Although the technology required to reliably detect HIV-1 DRMs on a POC platform is challenging, it would have widespread applications for the POC detection of critical drug-resistance, vaccine-escape, and gain-of-function mutations in other rapidly evolving epidemic viruses.

## Supporting Information

S1 Table Prevalence of Nucleoside RT Inhibitor (NRTI) Drug-Resistance Mutations (DRMs) in Antiretroviral (ARV)-Naïve and -Treated Individuals and Their Estimated Contributions to Reduced NRTI Susceptibility. (DOCX)

S2 Table Prevalence of Non-Nucleoside RT Inhibitor (NNRTI) Drug-Resistance Mutations (DRMs) in Antiretroviral (ARV)-Naïve and -Treated Individuals and Their Estimated Contributions to Reduced NNRTI Susceptibility. (DOCX)

S3 Table Prevalence of Protease Inhibitor (PI) Drug-Resistance Mutations (DRMs) in PI-Naïve and -Treated Individuals and Their Estimated Contributions to Reduced PI Susceptibility. (DOCX)

S4 Table Summary of Sequences from Individuals Receiving NRTI/NNRTI First-Line Regimens. (DOCX)

S5 Table Absolute and Cumulative Percent of Each Major Nucleoside (NRTI) Drug-Resistance Mutation (DRM) in 467 Individuals with Virological Failure and Intermediate or High-Level Acquired NRTI Drug Resistance while Receiving a First-Line TDF Containing Regimen. (DOCX)

S6 Table Absolute and Cumulative Percent of Each Major Nucleoside (NRTI) Drug-Resistance Mutation (DRM) in 712 Children with Virological Failure and Intermediate or High-Level Acquired NRTI Drug Resistance while Receiving a First-Line NRTI/NNRTI Regimen. (DOCX)

S7 Table Absolute and Cumulative Percent of Each Major Nonnucleoside (NNRTI) Drug-Resistance Mutation (DRM) in 721 Children with Virological Failure and Intermediate or High-Level Acquired NNRTI Drug Resistance while Receiving a First-Line NRTI/NNRTI Regimen. (DOCX)

S8 Table Proportion of Different Codons at Position 103 in HIV-1 RT Sequences from 26,358 Individuals in the Stanford HIV Drug Resistance Database From Six Low- and Middle-Income Country Regions. (DOCX)

S9 Table Proportion of Different 24 Nucleotide Sequences Flanking Position 103 in HIV-1 RT Sequences from 26,358 Individuals in the Stanford HIV Drug Resistance Database From Six Low- and Middle-Income Country Regions. (DOCX)
