# Supplementary material for: HIV-1 Drug Resistance Mutations: Potential Applications for Point-of-Care Genotypic Resistance Testing
Source: PLoS One. 2015 Dec 30;10(12):e0145772. doi: 10.1371/journal.pone.0145772 (PMC4696791; doi:10.1371/journal.pone.0145772)
Supplement: S1 Table — (DOCX) [file pone.0145772.s001.docx]

**S1 Table. Prevalence of Nucleoside RT Inhibitor (NRTI) Drug-Resistance Mutations (DRMs) in Antiretroviral (ARV)-Naïve and -Treated Individuals and Their Estimated Contributions to Reduced NRTI Susceptibility**

| DRM | HIVDB  Score*^a^* | SDRM*^b^* | Prevalence (%)*^c^* | |  | Phenotypic Fold Resistance*^d^* | | | |
| --- | --- | --- | --- | --- | --- | --- | --- | --- | --- |
|  |  |  | ARV-Naïve  (n=54,728) | ARV-Treated  (n=25,424) |  | 3TC  (n=1361) | ABC  (n=1267) | AZT  (n=1373) | TDF  (n=1081) |
| [M184V](javascript:%20void(0)) | 60 | + | 0.2 | 52 |  | **>50** | **3** | 0.3 | 0.5 |
| [K65R](javascript:%20void(0)) | 60 | + | 0.04 | 4 |  | **5** | **3** | 0.8 | **2** |
| [Q151M](javascript:%20void(0)) | 60 | + | 0 | 3 |  | 1.7 | **4** | **5** | 1.1 |
| [M184I](javascript:%20void(0)) | 60 | + | 0.03 | 2 |  | **>50** | **1.7** | 0.3 | 0.6 |
| [T215Y](javascript:%20void(0)) | 45 | + | 0.02 | 28 |  | 1.5 | **1.8** | **6** | 1.4 |
| [T215F](javascript:%20void(0)) | 45 | + | 0.01 | 10 |  | 1.5 | **1.7** | **8** | **1.6** |
| [Y115F](javascript:%20void(0)) | 45 | + | 0.01 | 2 |  | 1.4 | **3** | **4** | 1.**7** |
| [T69i](javascript:%20void(0)) | 45 | + | 0 | 1 |  | **3** | **5** | **18** | **4** |
| [K70R](javascript:%20void(0)) | 30 | + | 0.07 | 18 |  | 1.3 | 1.3 | **5** | **1.7** |
| [L74V](javascript:%20void(0)) | 30 | + | 0.01 | 9 |  | 1 | **1.5** | 0.3 | 0.6 |
| [L74I](javascript:%20void(0)) | 30 | + | 0.02 | 4 |  | 0.8 | 1.2 | 0.8 | 0.8 |
| [D67d](javascript:%20void(0)) | 30 |  | 0 | 0.09 |  | NA | NA | NA | NA |
| [M41L](javascript:%20void(0)) | 15 | + | 0.3 | 30 |  | 1.1 | 1.1 | **2** | **1.5** |
| [D67N](javascript:%20void(0)) | 15 | + | 0.04 | 28 |  | 1.2 | 1.2 | **2** | 1.2 |
| [L210W](javascript:%20void(0)) | 15 | + | 0.06 | 19 |  | 1.2 | 1.4 | **4** | **1.6** |
| [T215I](javascript:%20void(0)) | 15 | + | 0.03 | 1 |  | 1.8 | **1.5** | **5** | **1.6** |
| [T215S](javascript:%20void(0)) | 15 | + | 0.3 | 0.9 |  | 0.8 | 0.8 | 0.3 | 0.7 |
| [T215C](javascript:%20void(0)) | 15 | + | 0.09 | 0.8 |  | 0.9 | 1 | 1.2 | 0.8 |
| [T215D](javascript:%20void(0)) | 15 | + | 0.3 | 0.6 |  | 1.3 | 0.8 | 0.3 | 0.6 |
| [T215V](javascript:%20void(0)) | 15 | + | 0.01 | 0.6 |  | 1.1 | 1 | 1.7 | 1 |
| [K70E](javascript:%20void(0)) | 15 | + | 0.02 | 0.6 |  | 2 | 1.1 | 0.2 | 1 |
| [K70G](javascript:%20void(0)) | 15 |  | 0 | 0.4 |  | 1.4 | 1.2 | 0.3 | 1.1 |
| [T69d](javascript:%20void(0)) | 15 |  | 0 | 0.2 |  | NA | NA | NA | NA |
| [T215E](javascript:%20void(0)) | 15 | + | 0.1 | 0.2 |  | 2 | 1.1 | 1.2 | 1.3 |
| [K65N](javascript:%20void(0)) | 15 |  | 0.03 | 0.1 |  | NA | NA | NA | NA |
| [K219Q](javascript:%20void(0)) | 10 | + | 0.09 | 11 |  | 1.1 | 1 | 0.9 | 1 |
| [T69D](javascript:%20void(0)) | 10 | + | 0.03 | 6 |  | 1.1 | 1 | 0.8 | 0.9 |
| [K219E](javascript:%20void(0)) | 10 | + | 0.03 | 6 |  | 1 | 0.9 | 0.4 | 0.8 |
| [V75M](javascript:%20void(0)) | 10 | + | 0.03 | 3 |  | 0.9 | 1.3 | 1.4 | 1.1 |
| [K219N](javascript:%20void(0)) | 10 | + | 0.04 | 3 |  | 1.2 | 1.1 | 1.1 | -1 |
| [K219R](javascript:%20void(0)) | 10 | + | 0.07 | 3 |  | 1.8 | **1.6** | **3** | **1.5** |
| [D67G](javascript:%20void(0)) | 10 | + | 0.05 | 2 |  | 1.1 | 1.1 | **2** | 1.2 |
| [F116Y](javascript:%20void(0)) | 10 | + | 0.01 | 2 |  | 1.1 | 1 | **3** | 1.2 |
| [F77L](javascript:%20void(0)) | 10 | + | 0.1 | 2 |  | 1 | 0.9 | **4** | 1.4 |
| [V75T](javascript:%20void(0)) | 10 | + | 0 | 1 |  | 1.8 | **1.6** | 0.8 | 0.9 |
| [D67E](javascript:%20void(0)) | 10 | + | 0.01 | 0.6 |  | 1.2 | **1.5** | 1 | **1.5** |
| [K70T](javascript:%20void(0)) | 10 |  | 0.03 | 0.3 |  | 0.1 | 0.9 | **3** | **1.8** |
| [K70N](javascript:%20void(0)) | 10 |  | 0.03 | 0.3 |  | 1 | 1.3 | 1.6 | 1.4 |
| [K70Q](javascript:%20void(0)) | 10 |  | 0.02 | 0.2 |  | NA | NA | NA | NA |
| [K65E](javascript:%20void(0)) | 10 |  | 0.03 | 0.1 |  | NA | NA | NA | NA |

*^a^*HIVDB Score: Highest penalty score according to the Stanford HIV Drug Resistance Database (HIVDB) genotypic resistance interpretation program (version 7.0) for lamivudine (3TC), abacavir (ABC), zidovudine (AZT), and tenofovir (TDF). Scores of 15 to 29, 30 to 59, and ≥60 indicate low, intermediate, and high-level resistance. Emtricitabine (FTC) and 3TC scores are identical. *^b^*Surveillance Drug Resistance Mutation (SDRM): In ARV-naïve individuals, these DRMs are indicators of transmitted drug resistance (TDR) [1]. *^c^*DRM prevalence in samples from individuals with known ARV treatment history in HIVDB. The ARV-Naïve category excludes viruses with ≥2 SDRMs considered to be consistent with TDR rather than natural variation. *^d^*Estimated contribution to fold-reduced susceptibility based on linear regression analysis of PhenoSense susceptibility test results [2] (http://hivdb.stanford.edu/pages/genopheno.dataset.html). ‘NA’: fewer than three phenotypes with the DRM. Fold-resistance levels in bold (≥1.5 for ABC and 3TC, ≥2 for AZT, and ≥3 for 3TC) indicate a statistically and probable clinically significant increase above 1.0 compared with wildtype.

**References**

1. Bennett DE, Camacho RJ, Otelea D, Kuritzkes DR, Fleury H, Kiuchi M, et al. Drug resistance mutations for surveillance of transmitted HIV-1 drug-resistance: 2009 update. PLoS One. 2009;4(3):e4724. Epub 2009/03/07. doi: 10.1371/journal.pone.0004724. PubMed PMID: 19266092; PubMed Central PMCID: PMC2648874.

2. Petropoulos CJ, Parkin NT, Limoli KL, Lie YS, Wrin T, Huang W, et al. A novel phenotypic drug susceptibility assay for human immunodeficiency virus type 1. Antimicrob Agents Chemother. 2000;44(4):920-8. Epub 2000/03/18. PubMed PMID: 10722492; PubMed Central PMCID: PMC89793.
