# Supplementary material for: HIV-1 Drug Resistance Mutations: Potential Applications for Point-of-Care Genotypic Resistance Testing
Source: PLoS One. 2015 Dec 30;10(12):e0145772. doi: 10.1371/journal.pone.0145772 (PMC4696791; doi:10.1371/journal.pone.0145772)
Supplement: S2 Table — (DOCX) [file pone.0145772.s002.docx]

**S2 Table. Prevalence of Non-Nucleoside RT Inhibitor (NNRTI) Drug-Resistance Mutations (DRMs) in Antiretroviral (ARV)-Naïve and -Treated Individuals and Their Estimated Contributions to Reduced NNRTI Susceptibility**

| DRM | HIVDB  Score*^a^* | SDRM*^b^* | Prevalence (%)*^c^* | |  | Phenotypic Fold Resistance*^d^* | | | |
| --- | --- | --- | --- | --- | --- | --- | --- | --- | --- |
|  |  |  | ARV-Naïve  (n=54,728) | ARV-Treated  (n=25,424) |  | NVP  (n=1694) | EFV  ( n=1687) | ETR  (n=484) | RPV  (n=183) |
| [K103N](javascript:%20void(0)) | 60 | + | 1.0 | 36 |  | **24** | **21** | 1.3 | 2 |
| [Y181C](javascript:%20void(0)) | 60 | + | 0.1 | 20 |  | **16** | **2** | **8** | **3** |
| [G190A](javascript:%20void(0)) | 60 | + | 0.2 | 15 |  | **11** | **11** | 0.9 | 1.3 |
| [V106M](javascript:%20void(0)) | 60 | + | 0.01 | 5 |  | **18** | **32** | 0.6 | NA |
| [L100I](javascript:%20void(0)) | 60 | + | 0.01 | 4 |  | **3** | **14** | **6** | **7** |
| [Y188L](javascript:%20void(0)) | 60 | + | 0.04 | 4 |  | **>50** | **>50** | **3** | **10** |
| [G190S](javascript:%20void(0)) | 60 | + | 0.01 | 3 |  | **35** | **>50** | 0.9 | NA |
| [M230L](javascript:%20void(0)) | 60 | + | 0.02 | 2 |  | **6** | **7** | **4** | **5** |
| [V106A](javascript:%20void(0)) | 60 | + | 0.01 | 2 |  | **>50** | **7** | 0.4 | NA |
| [K103S](javascript:%20void(0)) | 60 | + | 0.04 | 2 |  | **11** | **7** | 1.5 | 1.7 |
| [K101P](javascript:%20void(0)) | 60 | + | 0 | 1 |  | **18** | **25** | **22** | **>50** |
| [Y188C](javascript:%20void(0)) | 60 | + | 0.01 | 0.9 |  | **>50** | **35** | NA | NA |
| [Y181I](javascript:%20void(0)) | 60 | + | 0.01 | 0.9 |  | **>50** | 1.4 | **30** | **24** |
| [Y181V](javascript:%20void(0)) | 60 | + | 0 | 0.6 |  | **>50** | 2 | **>50** | NA |
| [G190E](javascript:%20void(0)) | 60 | + | 0.02 | 0.5 |  | **>50** | **>50** | **>50** | **27** |
| [Y188H](javascript:%20void(0)) | 60 | + | 0.03 | 0.5 |  | **5** | **9** | NA | NA |
| [G190Q](javascript:%20void(0)) | 60 |  | 0 | 0.3 |  | **>50** | **>50** | NA | NA |
| [K101E](javascript:%20void(0)) | 30 | + | 0.2 | 8 |  | **2** | **3** | 1.5 | **2** |
| [A98G](javascript:%20void(0)) | 30 |  | 0.2 | 6 |  | **2** | **2** | 1.4 | **3** |
| [P225H](javascript:%20void(0)) | 30 | + | 0.02 | 4 |  | **2** | **3** | 1.2 | NA |
| [F227L](javascript:%20void(0)) | 30 |  | 0.04 | 3 |  | 1.4 | **2** | 2 | NA |
| [K238T](javascript:%20void(0)) | 30 |  | 0.04 | 2 |  | **3** | **2** | 1.4 | NA |
| [Y318F](javascript:%20void(0)) | 30 |  | 0.1 | 2 |  | NA | NA | NA | NA |
| [E138K](javascript:%20void(0)) | 30 |  | 0.1 | 0.4 |  | -0.6 | 1 | 2 | **1.6** |
| [F227C](javascript:%20void(0)) | 30 |  | 0 | 0.04 |  | NA | NA | NA | NA |
| [N348I](javascript:%20void(0)) | 15 |  | 0.09 | 14 |  | NA | NA | NA | NA |
| [V108I](javascript:%20void(0)) | 15 |  | 0.5 | 9 |  | **2** | **3** | 1 | 0.9 |
| [E138A](javascript:%20void(0)) | 15 |  | 3 | 3 |  | 1.5 | 1.6 | 2 | **1.8** |
| [K101H](javascript:%20void(0)) | 15 |  | 0 | 1 |  | **3** | **3** | 1.3 | 1 |
| [E138Q](javascript:%20void(0)) | 15 |  | 0.03 | 1 |  | 1.4 | 1 | NA | NA |
| [E138G](javascript:%20void(0)) | 15 |  | 0.3 | 0.7 |  | **2** | 1.4 | **3** | **1.7** |
| [V179F](javascript:%20void(0)) | 15 | + | 0 | 0.3 |  | 0.9 | **3** | **3** | 0.4 |
| [V179D](javascript:%20void(0)) | 10 |  | 2 | 3 |  | **3** | **5** | **3** | **1.8** |

*^a^*HIVDB Score: The highest mutation penalty score according to the Stanford HIV Drug Resistance Database (HIVDB) genotypic resistance interpretation program (version 7.0) for nevirapine (NVP), efavirenz (EFV), etravirine (ETR), and rilpivirine (RPV). Total scores of 15 to 29, 30 to 59, and ≥60 indicates low-level, intermediate, and high-level resistance. *^b^*Surveillance Drug Resistance Mutation (SDRM): When present in ARV-naïve individuals, these DRMs are considered specific indicators of transmitted drug resistance (TDR) [1]. *^c^*Prevalence of DRM in samples from individuals with known ARV treatment history in HIVDB. The ARV-Naïve category excludes viruses containing ≥2 SDRMs as these were considered to be consistent with TDR rather than natural variation. Nonetheless, the 1.0% prevalence of K103N in ARV-naïve individuals reflects its common occurrence in individuals with TDR.. *^d^*Estimated contribution to fold-reduced susceptibility based on linear regression analysis of PhenoSense susceptibility test results [2] (http://hivdb.stanford.edu/pages/genopheno.dataset.html). ‘NA’: fewer than three phenotypes with the DRM. Fold-resistance levels in bold (≥2 for NVP, EFV, and NVP and ≥3 for ETR) indicate a statistically and probable clinically significant increase above 1.0 compared with wildtype.

**References**

1. Bennett DE, Camacho RJ, Otelea D, Kuritzkes DR, Fleury H, Kiuchi M, et al. Drug resistance mutations for surveillance of transmitted HIV-1 drug-resistance: 2009 update. PLoS One. 2009;4(3):e4724. Epub 2009/03/07. doi: 10.1371/journal.pone.0004724. PubMed PMID: 19266092; PubMed Central PMCID: PMC2648874.

2. Petropoulos CJ, Parkin NT, Limoli KL, Lie YS, Wrin T, Huang W, et al. A novel phenotypic drug susceptibility assay for human immunodeficiency virus type 1. Antimicrob Agents Chemother. 2000;44(4):920-8. Epub 2000/03/18. PubMed PMID: 10722492; PubMed Central PMCID: PMC89793.
