# Supplementary material for: HIV-1 Drug Resistance Mutations: Potential Applications for Point-of-Care Genotypic Resistance Testing
Source: PLoS One. 2015 Dec 30;10(12):e0145772. doi: 10.1371/journal.pone.0145772 (PMC4696791; doi:10.1371/journal.pone.0145772)
Supplement: S4 Table — (DOCX) [file pone.0145772.s004.docx]

**S4 Table. Summary of Sequences from Individuals Receiving NRTI/NNRTI First-Line Regimens**

| Regimen | No. Pts | No. Refs | Subtype % | | | | | | | | |
| --- | --- | --- | --- | --- | --- | --- | --- | --- | --- | --- | --- |
|  |  |  | A | B | C | 01 | 02 | D | G | F | Others |
| AZT/3TC/EFV | 644 | 53 | 14.8 | 21.9 | 32.6 | 8.7 | 6.5 | 3.6 | 9.9 | 1.2 | 0.8 |
| D4T/3TC/EFV | 1318 | 44 | 2.1 | 6 | 86.7 | 2.4 | 0.9 | 0.3 | 0.6 | 0.3 | 0.6 |
| TDF/3TC/EFV | 251 | 16 | 2 | 15.9 | 72.9 | 0.4 | 4 | 0 | 4.4 | 0.4 | 0 |
| TDF/FTC/EFV | 349 | 11 | 6 | 39.3 | 20.9 | 8.6 | 5.2 | 2 | 15.5 | 1.4 | 1.1 |
| ABC/3TC/EFV | 113 | 9 | 5.3 | 13.3 | 75.2 | 0 | 2.7 | 0 | 3.5 | 0 | 0 |
| AZT/3TC/NVP | 673 | 54 | 17.8 | 8.9 | 25 | 10.1 | 13.7 | 9.1 | 11.9 | 0.7 | 2.8 |
| D4T/3TC/NVP | 1385 | 50 | 6.1 | 2.7 | 25.1 | 38.7 | 11.5 | 2.4 | 8.7 | 0.8 | 4 |
| TDF/3TC/NVP | 85 | 12 | 2.4 | 11.8 | 45.9 | 3.5 | 18.8 | 2.4 | 15.3 | 0 | 0 |
| TDF/FTC/NVP | 87 | 9 | 9.2 | 31 | 16.1 | 6.9 | 8 | 3.4 | 25.3 | 0 | 0 |
| ABC/3TC/NVP | 21 | 7 | 19 | 33.3 | 4.8 | 4.8 | 19 | 0 | 19 | 0 | 0 |
| *Total* | 4926 | 95 | 7.6 | 11.2 | 46 | 14.9 | 7.4 | 2.7 | 7.7 | 0.7 | 1.8 |

Abbreviations: AZT (zidovudine), 3TC (lamivudine), EFV (efavirenz), D4T (stavudine), TDF (tenofovir), FTC (emtricitabine), ABC (abacavir), NVP (nevirapine), 01 (CRF01_AE), 02 (CRF02_AG).
