# Supplementary material for: HIV-1 Drug Resistance Mutations: Potential Applications for Point-of-Care Genotypic Resistance Testing
Source: PLoS One. 2015 Dec 30;10(12):e0145772. doi: 10.1371/journal.pone.0145772 (PMC4696791; doi:10.1371/journal.pone.0145772)
Supplement: S5 Table — (DOCX) [file pone.0145772.s005.docx]

**S5 Table. Absolute and Cumulative Percent of Each Major Nucleoside (NRTI) Drug-Resistance Mutation (DRM)** **in 467 Individuals with Virological Failure and Intermediate or High-Level Acquired NRTI Drug Resistance while Receiving a First-Line TDF Containing Regimen***^a^*

| DRM | Absolute %*^b^* | Cumulative %*^c^* |
| --- | --- | --- |
| M184V | 72.6 | 72.6 |
| K65R | 48 | 93.4 |
| M184I | 13.7 | 98.7 |
| Y115F | 11.6 | 99.6 |
| Q151M | 0.9 | 99.8 |
| T215Y | 1.9 | 100 |
| L74I | 6.2 | 100 |
| L74V | 4.9 | 100 |
| K70R | 4.7 | 100 |
| T215F | 1.7 | 100 |

*^a^*NRTI DRM with an HIVDB score ≥30.

*^b^*Absolute %: number of individuals with DRM / number of individuals with intermediate or high-level NRTI resistance.

*^c^*Cumulative %: number of individuals with one or more of the preceding DRMs in the list / number of individuals with intermediate or high-level NRTI resistance.
