# Supplementary material for: HIV-1 Drug Resistance Mutations: Potential Applications for Point-of-Care Genotypic Resistance Testing
Source: PLoS One. 2015 Dec 30;10(12):e0145772. doi: 10.1371/journal.pone.0145772 (PMC4696791; doi:10.1371/journal.pone.0145772)
Supplement: S6 Table — (DOCX) [file pone.0145772.s006.docx]

**S6 Table. Absolute and Cumulative Percent of Each Major Nucleoside (NRTI) Drug-Resistance Mutation (DRM)** **in 712 Children with Virological Failure and Intermediate or High-Level Acquired NRTI Drug Resistance while Receiving a First-Line NRTI/NNRTI Regimen***^a^*

| DRM | Absolute %*^b^* | Cumulative %*^c^* |
| --- | --- | --- |
| M184V | 94.8 | 94.8 |
| K65R | 5.6 | 97.9 |
| M184I | 2.7 | 99.6 |
| K70R | 10 | 99.9 |
| T215Y | 6.7 | 100 |
| T215F | 8 | 100 |
| L74V | 8 | 100 |
| Y115F | 3.9 | 100 |
| Q151M | 3.5 | 100 |
| L74I | 2 | 100 |

*^a^*NRTI DRM with an HIVDB score ≥30.

*^b^*Absolute %: number of individuals with DRM / number of individuals with intermediate or high-level NRTI resistance.

*^c^*Cumulative %: number of individuals with one or more of the preceding DRMs in the list / number of individuals with intermediate or high-level NRTI resistance.
