# Supplementary material for: HIV-1 Drug Resistance Mutations: Potential Applications for Point-of-Care Genotypic Resistance Testing
Source: PLoS One. 2015 Dec 30;10(12):e0145772. doi: 10.1371/journal.pone.0145772 (PMC4696791; doi:10.1371/journal.pone.0145772)
Supplement: S7 Table — (DOCX) [file pone.0145772.s007.docx]

| **S7 Table. Absolute and Cumulative Percent of Each Major Nonnucleoside (NNRTI) Drug-Resistance Mutation (DRM)** **in 721 Children with Virological Failure and Intermediate or High-Level Acquired NNRTI Drug Resistance while Receiving a First-Line NRTI/NNRTI Regimen***^a^* |
| --- |

| DRM | Absolute %*^b^* | Cumulative %*^c^* |
| --- | --- | --- |
| K103N | 49.9 | 49.9 |
| V106M | 28.3 | 69.6 |
| Y181C | 21.5 | 84.9 |
| G190A | 17.6 | 90.8 |
| Y188L | 6.8 | 94.7 |
| G190S | 2.1 | 96.4 |
| G190Q | 1.2 | 97.6 |
| G190E | 1 | 98.5 |
| Y181V | 0.7 | 99 |
| M230L | 6.1 | 99.4 |
| V106A | 0.6 | 99.7 |
| L100I | 3.9 | 99.9 |
| Y188C | 2.2 | 100 |
| K103S | 3.3 | 100 |
| K101P | 2.5 | 100 |
| Y188H | 1.1 | 100 |

*^a^*NNRTI DRM with an HIVDB score ≥60.

*^b^*Absolute %: number of individuals with DRM / number of individuals with intermediate or high-level NNRTI resistance.

*^c^*Cumulative %: number of individuals with one or more of the preceding DRMs in the list / number of individuals with intermediate or high-level NNRTI resistance.
