# Supplementary material for: HIV-1 Drug Resistance Mutations: Potential Applications for Point-of-Care Genotypic Resistance Testing
Source: PLoS One. 2015 Dec 30;10(12):e0145772. doi: 10.1371/journal.pone.0145772 (PMC4696791; doi:10.1371/journal.pone.0145772)
Supplement: S8 Table — (DOCX) [file pone.0145772.s008.docx]

**S8 Table. Proportion of Different Codons at Position 103 in HIV-1 RT Sequences from 26,358 Individuals in the Stanford HIV Drug Resistance Database From Six Low- and Middle-Income Country Regions^*^**

| **Codon** | **Amino Acid** | **No. Sequences (%)** |
| --- | --- | --- |
| AAA | K | 22470 (85.2) |
| AAC | N | 1960 (7.4) |
| AAG | K | 1060 (4.0) |
| AAT | N | 449 (1.7) |
| AGA | R | 259 (1.0) |
| AGC | S | 86 (0.3) |
| AGG | R | 25 (0.1) |
| AGT | S | 18 (0.1) |

**^*^**Southern Africa, East Africa, West Africa, Central Africa, India, and the low- and middle-income countries in South and Southeast Asia. Eight codons occurring in ≥ 0.1% of individuals are shown. Overall, these 8 codons comprise 99.8% of all codons in this dataset.
