# Supplementary material for: HIV-1 Drug Resistance Mutations: Potential Applications for Point-of-Care Genotypic Resistance Testing
Source: PLoS One. 2015 Dec 30;10(12):e0145772. doi: 10.1371/journal.pone.0145772 (PMC4696791; doi:10.1371/journal.pone.0145772)
Supplement: S9 Table — (DOCX) [file pone.0145772.s009.docx]

**S9 Table. Proportion of Different 24 Nucleotide Sequences Flanking Position 103 in HIV-1 RT Sequences from 26,358 Individuals in the Stanford HIV Drug Resistance Database From Six Low- and Middle-Income Country Regions***

| **Flanking Sequence^†^** | **No. Sequences (%)** |
| --- | --- |
| GGGTTAAAAAAG‡‡‡AAATCAGTGACA | 4569 (19.1) |
| --T---------‡‡‡--------A--- | 4003 (16.7) |
| ------------‡‡‡--------A--- | 2327 (9.7) |
| --A---------‡‡‡--------A--- | 1915 (8.0) |
| --A---------‡‡‡------------ | 947 (4.0) |
| ---C--------‡‡‡--------A--- | 742 (3.1) |
| --CC--------‡‡‡--------A--- | 570 (2.4) |
| --C---------‡‡‡--------A--- | 471 (2.0) |
| ------------‡‡‡-----T--A--- | 353 (1.5) |
| ------------‡‡‡------A----- | 322 (1.3) |
| --TC--------‡‡‡--------A--- | 313 (1.3) |
| -----G------‡‡‡------------ | 244 (1.0) |

**^*^**Southern Africa, East Africa, West Africa, Central Africa, India, and the low- and middle-income countries in South and Southeast Asia. 12 flanking sequences occurring in ≥ 1.0% of individuals are shown. Overall, these 12 sequences comprise 70.1% of all codons in this dataset. **^†^**The three nucleotides comprising codon 103 are indicated by “‡‡‡”. Each of the nucleotides is indicated for the most commonly occurring pattern of 24 flanking nucleotides. For the remaining flanking sequences only the differences from the most commonly occurring pattern are shown.
